# Conditional Relative Survival of Ovarian Cancer: A Korean National Cancer Registry Study

**DOI:** 10.3389/fonc.2021.639839

**Published:** 2021-04-28

**Authors:** Dong Wook Shin, Jaeman Bae, Johyun Ha, Kyu-Won Jung

**Affiliations:** ^1^ Supportive Care Center/Department of Family Medicine, Samsung Medical Center, Sungkyunkwan University School of Medicine, Seoul, South Korea; ^2^ Department of Clinical Research Design & Evaluation, Samsung Advanced Institute for Health Science & Technology (SAIHST), Sungkyunkwan University, Seoul, South Korea; ^3^ Department of Obstetric and Gynecology, Hanyang University, Seoul, South Korea; ^4^ The Korea Central Cancer Registry, National Cancer Center, Goyang, South Korea; ^5^ Division of Cancer Registration and Surveillance, National Cancer Control Institute, National Cancer Center, Goyang, South Korea

**Keywords:** ovarian cancer, relative survival, conditional survival, Korea, cohort study

## Abstract

**Objective:**

Conditional relative survival (CRS) rates, which take into account changes in prognosis over time, are useful estimates for survivors and their clinicians as they make medical and personal decisions. We aimed to present the 5-year relative conditional survival probabilities of patients diagnosed with ovarian cancer from 1997–2016.

**Methods:**

This nationwide retrospective cohort study used data from the Korean Central Cancer Registry. Patients diagnosed with ovarian cancer between 1997 and 2016 were included. CRS rates were calculated stratified by age at diagnosis, cancer stage, histology, treatment received, year of diagnosis, and social deprivation index.

**Results:**

The 5-year relative survival rate at the time of diagnosis was 61.1% for all cases. The probability of surviving an additional 5 years, conditioned on having already survived 1, 2, 3, 4, and 5 years after diagnosis was 65.0, 69.5, 74.6, 79.3, and 83.9%, respectively. Patients with poorer initial survival estimates (older, distant stage, serous histology) generally showed the largest increases in CRS over time. The probability of death was highest in the first year after diagnosis (11.8%), and the conditional probability of death in the 2^nd^, 3^rd^, 4^th^, and 5^th^ years declined to 9.4%, 7.9%, 6.1%, and 5.2%, respectively.

**Conclusion:**

CRS rates for patients with ovarian cancer increased with each year they survived, but this did not reach the level of ‘no excess mortality’ even 5 years after diagnosis. The largest improvements in CRS were observed in patients with poorer initial prognoses. Our findings provide updated prognosis to ovarian cancer survivors and clinicians.

## Introduction

Ovarian cancer is the eighth most common cancer in women worldwide in terms of both incidence and mortality, accounting for approximately 295,000 cases and 185,000 deaths in 2018 (1). In Korea, the incidence of ovarian cancer has gradually increased, partly due to westernization of lifestyles and changes in reproductive factors (2). The age-standardized incidence increased from 5.0 in 1999 to 6.7 in 2016 (3). An estimated 2,832 cases of ovarian cancer occurred in Korea in 2019, comprising 2.8% of all newly diagnosed cancers in women that year (4). Ovarian cancer is the most common cause of gynecological cancer death in Korea, causing an estimated 1,271 deaths in 2019 (4).

Survival after ovarian cancer has improved significantly, although not dramatically: the five-year relative survival rate was 58.7% in 1993–1995 and 64.0% in 2012–2016 (3). Debulking surgery (including minimally-invasive approach), platinum-based chemotherapy, and most recently, targeted agents such as PARP inhibitors have driven that improvement (5–11). However, among the gynecologic malignancies, ovarian cancer has the poorest survival rate (12) because it is usually asymptomatic in the early stages and thus usually diagnosed at an advanced stage (~75% of cases) (13). Currently, no effective screening modality or nationwide screening program is available. The recurrence rate is also high, even after curative treatment.

Survival statistics are of great interest to cancer patients and their clinicians because they need to make important life and healthcare decisions based on those estimates. Currently, the most available survival estimate is the 5-year survival rate, i.e., the likelihood of surviving for 5 years from the time of diagnosis. However, the probability of surviving additional years generally improves significantly as time elapses after diagnosis, and patients and their clinicians thus need updated estimates to make informed decisions and plans at various timepoints (14).

Conditional survival (CS) is the most relevant indicator in this respect because it reflects how long patients have already survived after their cancer diagnosis. CS is a statistical method that describes the probability that a patient will survive a given additional amount of time (often 5 years) at various points after cancer diagnosis and reflects updated cancer prognoses as patients continue to survive. Relative survival (RS) is the ratio of observed survival to the expected survival of a general population. Conditional relative survival (CRS) consider changes in prognosis over time and therefore offer more useful estimates for survivors and their clinicians as they make medical and personal decisions.

Several cancer registry reported CS or CRS rates for ovarian cancer patients along with those for other major types of cancers (15–17). However, these studies were based on decades-old survival data (e.g. European 1985-2004; the US 1990-2001; and Japan 1993-2009). In addition, only the US Surveillance, Epidemiology, and End Results (SEER) studies reported CRS rates stratified by various factors including age, race, stage, histology, and grade) (17, 18); however, data for race were reported only reported as white, black, and others. Disparities might exist with regard to the race/ethnicity, and a recent SEER study showed slightly better survival among Asian/Pacific Island women (19). To the best of our knowledge, no such study has been performed using recent data or in an Asian country. The one Japanese study reported only overall CS estimates without any stratification according to patient factors (15).

In this study, we use Korean Central Cancer Registry (KCCR) data to present the 5-year CRS probabilities of patients diagnosed with ovarian cancer in 1997–2016, with follow up through 2017 for vital status. The effects of age, histology, stage, treatment received, year of diagnosis, and social deprivation index on the CRS estimates are also assessed.

## Methods

### Data Source and Study Population

The KCCR is a national cancer registry established and run by the Ministry of Health and Welfare in Korea. It was first launched as a hospital-based cancer registry in 1980 and was expanded into a nationwide, population-based, cancer registry in 1999. The completeness and quality of KCCR data are well documented in volumes IX (1999–2002) to XI (2008–2012) of *Cancer Incidence in Five Continents* (20). The completeness of the KCCR is estimated to be better than 98% [3]. The KCCR collects data on patient demographics, date of diagnosis, primary tumor site, histology according to the International Classification of Disease for Oncology Second Edition (ICD-O-3) (21), SEER stage at diagnosis, and treatment received during the 4 months after diagnosis. SEER stage information has been collected since 2005, and the information was complete **(** **Supplementary Table 1** **).**

Patients who were diagnosed with ovarian cancer as their first cancer between January 1, 1997, and December 31, 2016, were included in this study. Their vital status was linked to mortality data from the National Statistical Office of Korea. The last follow-up date was December 31, 2017. This study was approved by the institutional review board of the National Cancer Center in Korea (NCC 2020-0280), and the need for informed consent was waived as this study involved only deidentified data.

### Variables

Patients were categorized into 5 groups by age at cancer diagnosis: <40 years, 40–49 years, 50–59 years, 60–69 years, and ≥70 years. The stages at diagnosis (collected since January 1, 2005) were classified as localized, regional, distant, or unknown using the SEER staging scheme. Each SEER stage roughly corresponds to FIGO staging as follows: localized – FIGO IA and IB; regional – FIGO IC and II, and distant – FIGO III and IV (22). The histology of ovarian cancer was categorized as serous adenocarcinoma (ICD-O-3 morphology codes: 8050, 8441, 8450, 8460-8461), mucinous adenocarcinoma (ICD-O-3: 8470-8471, 8480-8482, 8490), endometrioid adenocarcinoma (ICD-O-3: 8380, 8382-8383, 8560, 8570), clear cell adenocarcinoma (ICD-O-3: 8005, 8310, 8443, 9110), and other, based on ICD-O-3 codes, previous literature (23), and the number of cases. Treatment information included receipt of surgery, chemotherapy, and radiotherapy during the first 4 months after diagnosis, but the intent of treatment (e.g. neoadjuvant, adjuvant, or palliative) was not specified, and treatment data after the first 4 months were not available. The years of diagnosis were classified using 5-year intervals: 1997–2001, 2002–2006, 2007–2011, and 2012–2016. Area-level socioeconomic disparity was evaluated using the Carstairs index (24) and categorized into quintiles, with the 1st quintile representing the wealthiest area.

### Statistical Analysis

RS approximates disease-specific survival and can overcome the limitation of inaccuracy in the cause-of-death data on death certificates (25). It is calculated as the observed survival among cancer patients divided by the expected survival of the general population of the same period, age, and sex (26). The population lifetables to calculate expected the mortality rate in the general population were obtained from Statistics Korea (Korean Statistical Information Service).

CRS is defined as the probability of surviving an additional y years on the condition that a patient has already survived x years. Therefore, the CRS for another y years is calculated by dividing the RS at (x + y) years by the RS at x years:

$$CS(y|x)=\frac{S(x+y)}{S(x)}$$

where S(x) is the RS at time x. For example, the 5-year CRS conditional on having already survived 3 years is calculated by dividing an 8-year cumulative RS by the 3-year cumulative RS. In this study, we present 5-year CRS rates conditioned on 1–5 years already survived after diagnosis. We calculated 95% confidence intervals [CIs] assuming that CS follows a normal distribution. Survival estimates were stratified by age group, SEER stage at diagnosis, histology, year of diagnosis, and deprivation index.

We also estimated the relative excess risk (RER) and its 95% CI to examine the relative effects of patients’ demographic and clinical characteristics on survival at baseline and different time periods already survived. Multivariate analyses included age, year of diagnosis, histology, deprivation, and stage at diagnosis. As SEER stage information was available from 2005 on, patients from 2005 were included in this analysis (N=18,336). RER was analyzed for patients who survived 2 years (N=13,106) and 5 years (N=6,587).

All analyses were conducted using Stata version 15.0 (StataCorp LP, College Station, TX) or SAS 9.4. All statistical tests were two-tailed, and *p-*values<0.05 were considered significant.

## Results

### Baseline Characteristics

**Table 1** summarizes the baseline characteristics of patients available for the calculation of RS and CRS at baseline, 2, and 5 years after baseline. A total of 25,859 patients with ovarian cancer diagnosed between 1997 and 2016 were identified from the KCCR registry, and the number of ovarian cancer cases per year increased during the study period. The most frequent age at diagnosis was 50–59, followed by 40–49 and 60–69.

**Table 1:** Baseline characteristics of Korean ovarian cancer patients, 1997–2016.

Variables		Number of patients	%	Patients available for CRS after year	
				2	5
	Total	25,859	100.0	18,756	10,894
Age					
	<40 years	3,908	15.1	3,265	2,427
	40–49 years	6,576	25.4	5,240	3,200
	50–59 years	7,318	28.3	5,540	2,999
	60–69 years	4,832	18.7	3,261	1,649
	≥70 years	3,225	12.5	1,450	619
Stage at diagnosis (since 2005, N=18,336)					
	Localized	4,902	26.7	4,180	2,622
	Regional	3,296	18.0	2,572	1,398
	Distant	8,899	48.5	5,489	2,062
	Unknown	1,239	6.8	865	505
Histology (ICD-O-3)					
	Serous adenocarcinoma	12,443	48.1	9,351	4,797
	Mucinous adenocarcinoma	4,178	16.2	3,291	2,360
	Endometrioid adenocarcinoma	2,391	9.2	1,963	1,345
	Clear cell adenocarcinoma	2,320	9.0	1,763	1,077
	Others^a)^	4,527	17.5	2,388	1,315
Treatment received^b)^					
	No treatment	1,414	5.5	588	388
	Surgery only	6,577	25.4	5,015	3,413
	Surgery + Chemotherapy	15,587	60.3	11,947	6,559
	Chemotherapy only	1,827	7.1	918	374
	Others^c)^	454	1.8	288	158
Year of diagnosis					
	1997–2001	4,431	17.1	3,249	2,509
	2002–2006	5,427	21.0	4,226	3,148
	2007–2011	7,138	27.6	5,644	4,229
	2012–2016	8,863	34.3	5,637	1,008
Deprivation index					
	1 (wealthiest)	10,716	41.4	8,015	4,666
	2	7,663	29.6	5,617	3,293
	3	3,227	12.5	2,245	1,259
	4	2,469	9.5	1,664	974
	5 (poorest)	1,784	6.9	1,215	702

^a)^Others included squamous cell carcinoma, transitional cell or Brenner carcinoma, mixed epithelial-stromal carcinoma, undifferentiated or other epithelial carcinoma. Histology other than type I and type II epithelial tumor was excluded from the study.

^b)^Treatment information is based on receipt of treatment within 4 months after diagnosis.

^c)^Others include radiotherapy only, chemotherapy + radiotherapy, surgery + radiotherapy, and surgery +chemotherapy + radiotherapy.

Serous adenocarcinoma accounted for around half (48.1%) of all ovarian cancer, followed by mucinous (16.2%), endometrioid (9.2%), and clear cell adenocarcinoma (9.0%). SEER stage information was available for the 18,336 patients diagnosed since 2005. Around half (48.5%) were diagnosed in the distant stage, and 18.0% and 26.7% were diagnosed in the regional and localized stages, respectively. Among all ovarian cancer patients, 41.4% and 29.6% of patients were from the wealthiest and second wealthiest areas, respectively.

The distribution of histology type by age group and the stage distribution by histologic type are described in **Supplementary Table 2** and **Supplementary Figure 1**, respectively. Overall, younger people had more non-serous type ovarian cancers (mucinous, endometrioid, and clear cell), and the localized stage was more common with non-serous types of ovarian cancer.

### Relative Survival and Conditional Relative Survival

**Figure 1:**
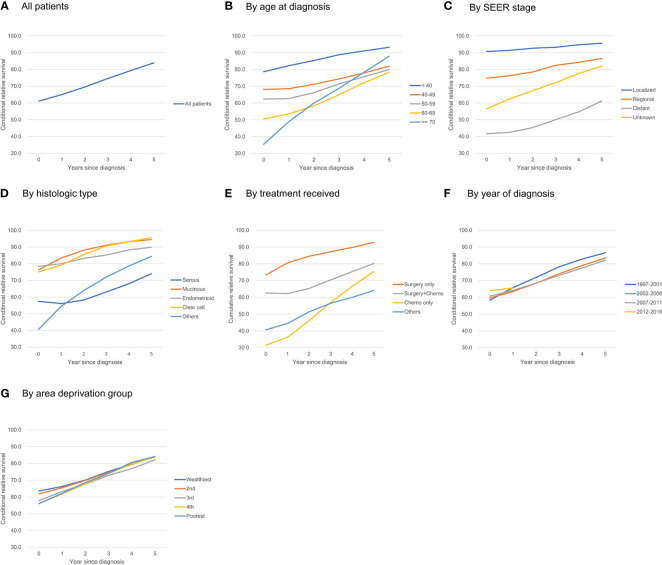
Five-year conditional relative survival of ovarian cancer patients. **(A)** all patients; **(B)** by age at diagnosis; **(C)** by SEER stage; **(D)** by histologic type; **(E)** by treatment received; **(F)** by year of diagnosis; **(G)** by area deprivation group.

For all ovarian patients, the 5-year and 10-year RS at the time of diagnosis were 61.1% and 51.1%, respectively. The probability of surviving an additional 5 years conditioned on having already survived 1, 2, 3, 4, and 5 years after diagnosis was 65.0, 69.5, 74.6, 79.3, and 83.9%, respectively **(** **Figure 1A** **)**. The 5-year CRS rates for all ovarian cancer patients and according to age group, stage, histology, treatment received, year of diagnosis, and social deprivation index are depicted in **Figure 1**. Detailed estimates are presented in **Table 2**.

**Table 2:** Relative and conditional survival (%), 1997–2016 (N=25,859).

			Relative survival (95% CI)					Conditional 5-year relative survival (95% CI)													
		N	5-year	95% CI	10-year	95% CI	N	1-year	95% CI	N	2-year	95% CI	N	3-year	95% CI	N	4-year	95% CI	N	5-year	95% CI
**Total**		25,859	61.1	(60.5 61.8)	51.1	(50.3 51.8)	22,809	65.0	(64.3 65.7)	18,756	69.5	(68.7 70.2)	15,560	74.6	(73.8 75.4)	12,983	79.3	(78.5 80.2)	10,894	83.9	(83.1 84.8)
**Age**																					
	< 40	3,908	78.7	(77.3 80.0)	73.3	(71.8 74.8)	3,643	82.3	(80.9 83.6)	3,265	85.3	(83.9 86.6)	2,947	88.8	(87.5 89.9)	2,648	91.1	(89.8 92.2)	2,427	93.2	(92.1 94.3)
	40-49	6,576	68.1	(66.9 69.3)	55.8	(54.3 57.2)	6,149	68.6	(67.2 69.8)	5,240	71.2	(69.8 72.5)	4,458	74.4	(72.9 75.8)	3,799	77.9	(76.4 79.4)	3,200	82.0	(80.4 83.5)
	50-59	7,318	62.4	(61.2 63.6)	49.8	(48.4 51.3)	6,769	62.7	(61.3 64.0)	5,540	66.2	(64.8 67.7)	4,525	71.4	(69.8 72.9)	3,680	75.7	(74.0 77.3)	2,999	80.0	(78.2 81.7)
	60-69	4,832	50.5	(48.9 52.1)	39.3	(37.5 41.1)	4,148	53.6	(51.8 55.4)	3,261	58.3	(56.3 60.3)	2,570	65.0	(62.7 67.2)	2,037	72.1	(69.7 74.5)	1,649	78.4	(75.7 80.9)
	>= 70	3,225	35.5	(33.4 37.5)	30.0	(27.4 32.7)	2,100	49.0	(46.1 52.0)	1,450	60.0	(56.2 63.6)	1,060	68.8	(64.3 73.1)	819	78.4	(73.2 83.4)	619	87.9	(81.7 93.5)
**Stage at diagnosis**																					
	Localized	4,902	90.7	(89.6 91.6)	86.3	(84.7 87.8)	4,779	91.4	(90.3 92.4)	4,180	92.7	(91.5 93.7)	3,643	93.2	(92.0 94.3)	3,071	94.7	(93.3 95.8)	2,622	95.6	(94.2 96.8)
	Regional	3,296	74.7	(72.9 76.4)	64.4	(61.8 66.8)	3,090	76.3	(74.4 78.1)	2,572	78.5	(76.4 80.4)	2,128	82.5	(80.4 84.5)	1,745	84.2	(81.7 86.4)	1,398	86.5	(83.7 89.0)
	Distant	8,899	41.7	(40.5 42.9)	25.5	(24.1 26.9)	7,448	42.5	(41.1 43.9)	5,489	45.3	(43.6 46.9)	4,008	50.1	(48.1 52.1)	2,933	54.7	(52.3 57.1)	2,062	61.2	(58.3 64.0)
	Unknown	1,239	56.6	(53.5 59.5)	46.2	(42.8 49.5)	1,047	62.5	(59.0 65.7)	865	67.3	(63.6 70.8)	724	72.2	(68.2 75.8)	597	77.6	(73.4 81.3)	505	82.0	(77.6 85.8)
**Histology**																					
	Serous adenocarcinoma	12,443	57.4	(56.4 58.4)	42.3	(41.2 43.4)	11,534	56.1	(55.0 57.1)	9,351	58.2	(57.0 59.4)	7,511	63.1	(61.8 64.3)	6,031	68.0	(66.6 69.4)	4,797	74.0	(72.4 75.5)
	Mucinous adenocarcinoma	4,178	76.3	(74.9 77.7)	71.8	(70.1 73.4)	3,754	83.4	(82.0 84.8)	3,291	88.1	(86.7 89.4)	2,908	91.1	(89.7 92.3)	2,580	93.2	(91.9 94.4)	2,360	94.5	(93.1 95.7)
	Endometrioid adenocarcinoma	2,391	78.4	(76.5 80.2)	70.2	(67.9 72.5)	2,242	80.0	(78.0 81.9)	1,963	83.2	(81.2 85.1)	1,742	85.2	(83.1 87.1)	1,524	88.3	(86.2 90.1)	1,345	89.9	(87.7 91.8)
	Clear cell adenocarcinoma	2,320	75.1	(73.1 77.0)	71.7	(69.4 73.9)	2,145	79.2	(77.1 81.2)	1,763	85.5	(83.5 87.4)	1,478	90.7	(88.7 92.4)	1,262	93.2	(91.2 94.8)	1,077	95.7	(93.7 97.2)
	Others	4,527	40.6	(39.1 42.2)	34.0	(32.4 35.7)	3,134	54.4	(52.4 56.4)	2,388	63.9	(61.7 66.1)	1,921	72.0	(69.6 74.4)	1,586	78.6	(76.0 81.0)	1,315	84.4	(81.7 86.8)
**Treatment received ^a)^**																					
	No treatment	1,414	32.8	(30.2 35.4)	27.7	(25.1 30.3)	704	60.0	(55.9 63.9)	588	68.2	(63.9 72.2)	499	76.3	(71.8 80.3)	437	81.8	(77.2 85.7)	388	85.0	(80.4 88.9)
	Surgery only	6,577	73.3	(72.1 74.5)	67.7	(66.2 69.0)	5,819	80.5	(79.2 81.6)	5,015	84.5	(83.2 85.7)	4,400	87.2	(85.9 88.4)	3,828	89.8	(88.5 91.0)	3,413	92.8	(91.5 94.0)
	Surgery+Chemo	15,587	62.6	(61.7 63.4)	50.0	(49.0 51.0)	14,548	62.2	(61.3 63.1)	11,947	65.3	(64.3 66.3)	9,785	70.4	(69.3 71.4)	8,045	75.3	(74.2 76.4)	6,559	80.2	(79.0 81.3)
	Chemo only	1,827	31.4	(29.0, 33.8)	23.5	(21.2 25.9)	1,367	36.2	(33.3 39.2)	918	46.1	(42.4 49.8)	648	56.9	(52.4 61.3)	485	66.6	(61.6 71.2)	374	75.4	(70.0 80.0)
	Others	454	40.6	(35.9 45.3)	25.9	(21.5 30.5)	371	44.4	(39.0 49.7)	288	51.3	(45.0 57.3)	228	56.5	(49.3 63.1)	188	60.0	(52.0 67.2)	158	64.1	(55.2 71.8)
**Year of diagnosis**																					
	1997-2001	4,431	58.3	(56.8 59.8)	50.3	(48.7 51.8)	3,743	65.7	(64.1 67.3)	3,249	71.9	(70.3 73.6)	2,908	78.2	(76.5 79.7)	2,687	82.8	(81.1 84.3)	2,509	86.6	(85.0 88.1)
	2002-2006	5,427	59.6	(58.3 61.0)	49.5	(48.1 50.9)	4,764	63.4	(62.0 64.8)	4,226	68.4	(66.9 69.8)	3,766	74.1	(72.6 75.6)	3,420	79.0	(77.5 80.4)	3,148	83.4	(81.9 84.8)
	2007-2011	7,138	60.7	(59.5 61.8)	49.6	(48.2 51.0)	6,325	64.1	(62.9 65.3)	5,644	68.5	(67.2 69.8)	5,083	73.1	(71.7 74.4)	4,629	77.5	(76.1 79.0)	4,229	82.1	(80.4 83.7)
	2012-2016	8,863	64.0	(62.6 65.4)	–	–	7,977	65.6	(63.4 67.7)	–	–	–	–	–	–	–	–	–	–	–	–
**deprivation index**																					
	1 (wealthiest)	10,716	63.6	(62.5 64.6)	53.3	(52.1 54.5)	9,639	66.3	(65.2 67.4)	8,015	70.1	(69.0 71.3)	6,670	75.2	(73.9 76.4)	5,559	79.7	(78.4 81.0)	4,666	84.2	(82.9 85.4)
	2	7,663	61.9	(60.7 63.1)	51.9	(50.5 53.3)	6,795	65.5	(64.1 66.7)	5,617	69.9	(68.5 71.3)	4,663	74.7	(73.3 76.2)	3,914	79.4	(77.9 80.9)	3,293	84.2	(82.6 85.7)
	3	3,227	57.8	(55.9 59.7)	47.3	(45.1 49.4)	2,763	63.3	(61.2 65.4)	2,245	67.7	(65.4 69.9)	1,844	72.9	(70.4 75.3)	1,534	76.9	(74.2 79.3)	1,259	82.2	(79.4 84.7)
	4	2,469	56.1	(53.9 58.2)	46.8	(44.3 49.2)	2,089	61.8	(59.4 64.2)	1,664	67.7	(65.0 70.2)	1,383	74.1	(71.3 76.8)	1,156	79.6	(76.6 82.3)	974	83.9	(80.8 86.6)
	5 (poorest)	1,784	56.1	(53.6 58.6)	47.0	(44.1 49.8)	1,523	62.2	(59.3 64.9)	1,215	68.6	(65.5 71.5)	1,000	74.1	(70.8 77.2)	820	80.6	(77.2 83.7)	702	84.2	(80.6 87.3)

^a)^Treatment information is based on receipt of treatment within 4 months after diagnosis.

Patients had a higher 5-year RS when they were diagnosed at younger ages: the 5-year RS for patients <40 years of age was 78.7%, and the 5-year RS for patients ≥70 years was 35.5%. The difference in the 5-year CRS became smaller over time, but it remained significant even after 5 years: the 5-year CRS for patients <40 years of age was 93.2%, whereas the 5-year CRS for patients 60–69 years was 78.4%. One exception was patients older than 70: they showed the lowest 5-year RS at diagnosis (35.5%), but as they survived for longer, their 5-year CRS increased significantly, reaching 87.9% and surpassing that of patients diagnosed at 40–69 years of age **(** **Figure 1B** **)**.

The improvement in the 5-year CRS was greatest for patients at the distant stage (41.7% at diagnosis to 61.2% after 5 years), whereas patients with regional (74.7% to 86.5%) or localized disease (90.7% to 95.6%) had a much smaller change in their survival probability over time (**Figure 1C**).

At diagnosis, the 5-year RS among patients with serous adenocarcinoma (57.4%) was lower than that for all other histological types: mucinous adenocarcinoma (76.3%), endometrioid adenocarcinoma (78.4%), clear cell adenocarcinoma (75.1%). The 5-year CRS for each histologic type increased over time; however, whereas the 5-year CRS rates at 5 years after diagnosis for clear cell, mucinous, and endometrioid adenocarcinoma reached 90% or more, that for serous adenocarcinoma reached only 74.0%. Thus, the disparity in RS did not decrease significantly (**Figure 1D**).

Patients who received surgery only showed high 5-year RS from the time of diagnosis (73.3%) and reached >90% after 5 years. Those who had surgery + chemotherapy showed 62.6% of 5-year RS at diagnosis, and it increased to 80.2% after 5 years. Those who received chemotherapy only showed poorest survival rate of 31.4% at diagnosis, but this rapidly increased and reached >95% when they survived for 5 years **(** **Figure 1E** **).**

The 5-year RS was higher for patients who were recently diagnosed; for example, the 5-year RS for those diagnosed in 2007–2011 was higher than that for those diagnosed in 1997–2001 (60.7% vs. 58.3%). However, after 1 year, the 5-year CRS for those groups crossed (64.1% vs. 65.7%), and the CRS for those diagnosed in 2007–2011 became lower than that of those diagnosed in 1997–2001 (82.1% vs. 86.6%) **(** **Figure 1F** **)**.

Patients living in deprived areas showed lower 5-year RS rates (63.6% in the wealthiest area vs. 56.1% in the most deprived area), but that gap decreased over time and disappeared 3–5 years after diagnosis **(** **Figure 1G** **)**.

### Conditional Probability of Death

**Figure 2:**
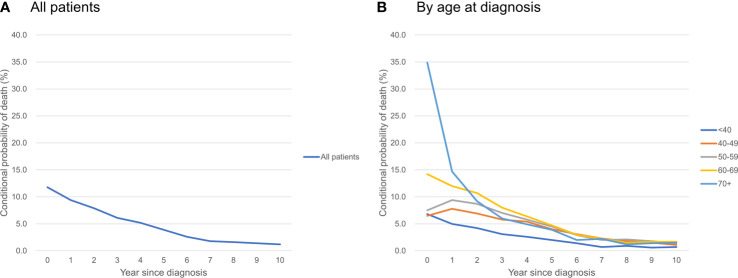
Conditional probability of death of ovarian cancer patients **(A)** all patients; **(B)** by age at diagnosis.

**Table 3** shows the conditional probability of all-cause death according to the year since diagnosis and age group. Older patients had a higher risk of all-cause death at the time of diagnosis, but it declined rapidly over time: patients who were ≥70 years of age had a very high probability of death in the first year after diagnosis (34.9%), but the conditional probability of death in the 2^nd^, 3^rd^, 4^th^, and 5^th^ years after diagnosis declined abruptly to 14.7, 9.2, 6.0, and 4.9%, respectively. In contrast, younger patients who were 40-59 years were at lower risk of death at the time of diagnosis, but their mortality risk increased in the 2^nd^ year and then declined only slowly over time **(** **Figure 2** **)**.

**Table 3:** Conditional probability of death by age group and year since diagnosis.

Year since diagnosis	All age group	Age group				
		<40	40-49	50-59	60-69	70+
0-1	11.8	6.8	6.5	7.5	14.2	34.9
1-2	9.4	5.0	7.8	9.4	12.0	14.7
2-3	7.9	4.2	6.9	8.7	10.7	9.2
3-4	6.1	3.1	5.8	7.0	8.0	6.0
4-5	5.2	2.6	5.4	5.8	6.4	4.9
5-6	3.9	2.0	4.0	4.5	4.7	3.9
6-7	2.6	1.4	3.1	2.9	3.0	2.0
7-8	1.8	0.7	2.3	2.0	2.2	2.2
8-9	1.6	0.9	1.8	2.1	1.5	1.2
9-10	1.4	0.6	1.5	1.8	1.7	1.4
10-11	1.2	0.7	1.1	1.4	1.7	1.5

### Effects of Baseline Characteristics on Mortality According to Time Already Survived Since Diagnosis

At baseline, patients in their 50’s showed the lowest RER (0.74, 95% CI 0.60–0.90), and patients in their 70’s showed the highest RER (2.90, 95% CI 2.41–3.50), suggesting a U-shaped pattern. However, 5 years after diagnosis, RER increased linearly with age. Diagnosis later in the study period was associated with low RER, regardless of time survived since diagnosis.

The effect of histology decreased with longer survival: RERs decreased from 3.27, 1.68, and 2.87 at baseline to 1.43, 0.83, 1.56 at 5 years after diagnosis for mucinous, endometrioid, and clear cell histology, respectively. RER did not differ significantly by SEER stage or social deprivation index according to the time survived since diagnosis (**Table 4**).

**Table 4:** Factors associated with mortality according to survival time since diagnosis, 2005–2016: multivariate analyses (N=18,336).

		At diagnosis	2 years after diagnosis	5 years after diagnosis
		RER (95% CI)	RER (95% CI)	RER (95% CI)
**N, included**		18,336	13,106	6,587
**Age**				
	< 40 years	1	1	1
	40–49 years	0.77 (0.63, 0.95)	0.91 (0.79, 1.06)	1.01 (0.90, 1.12)
	50–59 years	0.74 (0.60, 0.90)	0.94 (0.81, 1.09)	1.12 (1.00, 1.24)
	60–69 years	1.17 (0.96, 1.42)	1.26 (1.09, 1.46)	1.40 (1.26, 1.57)
	≥ 70 years	2.90 (2.41, 3.50)	2.86 (2.47, 3.30)	2.69 (2.41, 3.01)
**Year of diagnosis**				
	2005–2010	1	1	1
	2011–2016	0.83 (0.76, 0.91)	0.81 (0.75, 0.86)	0.83 (0.78, 0.87)
**Histology**				
	Serous	1	1	1
	Mucinous	3.27 (2.76, 3.86)	2.24 (1.98, 2.54)	1.43 (1.29, 1.58)
	Endometrioid	1.68 (1.31, 2.15)	1.19 (0.99, 1.42)	0.83 (0.72, 0.94)
	Clear Cell	2.87 (2.34, 3.52)	2.34 (2.03, 2.69)	1.56 (1.40, 1.74)
	Other	4.45 (3.97, 4.99)	3.10 (2.86, 3.37)	2.04 (1.92, 2.18)
**Deprivation index**				
	1 (wealthiest)	1	1	1
	2	1.12 (1.00, 1.26)	1.09 (1.00, 1.19)	1.07 (1.01, 1.14)
	3	1.18 (1.02, 1.36)	1.15 (1.03, 1.28)	1.08 (0.99, 1.17)
	4	1.11 (0.95, 1.30)	1.19 (1.06, 1.34)	1.14 (1.04, 1.25)
	5 (poorest)	1.10 (0.91, 1.33)	1.16 (1.01, 1.33)	1.19 (1.07, 1.32)
**Stage at diagnosis**				
	Localized	1	1	1
	Regional	3.61 (2.77, 4.78)	3.31 (2.77, 4.78)	3.16 (2.77, 4.78)
	Distant	9.72 (7.66, 12.46)	8.92 (7.66, 12.46)	9.00 (7.66, 12.46)
****	Unknown	6.36 (4.83, 8.42)	5.46 (4.83, 8.42)	5.20 (4.83, 8.42)

RER, relative excess risk; CI, confidence interval.

Multivariate analyses included all variables presented.

## Discussion

In this study using the nationwide population-based cancer registry of Korea, we demonstrated that the 5-year CRS in patients with ovarian cancer improves as they survive each additional year after their diagnosis, and patients who are older at diagnosis, have advanced-stage disease, or a serous histology showed larger CRS increases over time than other patients. The RER analyses showed that the prognostic importance of histology decreases as time already survived increases, whereas the prognostic importance of age, stage, year of diagnosis, and social deprivation index remains similar over time.

Overall, the survival rates following ovarian cancer among Korean patients are substantially higher than previous reports from other countries. The 5-year RS was 61.1% in our study, compared with 36.1% in Europe from 1995 to 2002 (27) and 45.0% in Canada from 2006-2008 (28). The 10-year RS in our study was 51.1%, compared with 43.9% among Japanese patients diagnosed between 1993 and 2009 (15). Our stage-specific 5-year RS rates for localized, regional, and distant disease were 92.7, 74.7, and 41.7%, whereas those rates were 92.5, 73.9, and 28.9% in the US SEER registry (29). Higher survival estimates in Korea might be due to earlier detection through the concomitant transvaginal ultrasound screening during the national cervical screening program and/or higher rate of optimal cytoreduction due to better access to gynecologic oncologists, but further investigation of differences between countries is needed **(** **Supplementary Table 3** **).** Indeed, a recent SEER study reported slightly better survival in Asian/Pacific island women than other ethnicities, and the disparity was largely attributed to dissimilarities in clinical care (19). Our 5-year CRS at 5 years after diagnosis (83.9%) is quite similar to the rates in the US SEER data and Japan (85.6%) (15).

Our data show that the 5-year CRS increases with time since diagnosis, indicating that the residual risk diminishes substantially over time. As with many cancers, the greatest number of deaths from ovarian cancer occurred during the first year following diagnosis (11.8%); the number of death then decreased to 9.4 in the second year, 7.9% in the third year. By 10 years after diagnosis, the number of all-cause death had decreased to 1.2%. Similar findings were observed also in the US SEER data (22, 30). Providing updated survival information to survivors could reduce their feelings of uncertainty and mitigate their fear of cancer recurrence (31).

Nonetheless, the 5-year CRS at 5 years after diagnosis with ovarian cancer was 83.8%, which is not close to the expected survival of age-matched controls in the general population. The 5-year CRS needs to exceed 95% to enable the survival of patients to be considered equivalent to that in the general population (16, 25). A previous US study also found that a 5-year CRS>95% was not reached within 10 years for ovarian cancer patients aged 45 and older (16). Thus, ovarian cancer patients continue to have excess risk of mortality compared to the general population. This could be due to late side effects of treatment, late recurrence, a second primary cancer, or comorbidity (25, 32). These ongoing reduced survival expectations for ovarian cancer patients have implications for their healthcare; they need regular surveillance and monitoring even after they have survived for several years after their diagnosis (25). Currently, there is no clear evidence to guide routine follow-up for ovarian cancer, especially after 5 years (33–35). For example, the interval for follow-up and whether it can be performed by general practitioners or nurse specialists continue to be debated. The CRS estimates from this study suggest the need for long-term follow-up in at least a subset of patients, and our data could be used to determine individualized long-term follow-up schedules.

Consistent with a previous study (16), we found that initial differences between age groups in RS at diagnosis diminish over time. Young survivors (<40 years) persistently showed higher CRS than older survivors, probably due to earlier detection and lower disease grade **(** **Supplementary Table 4** **)** (28, 36), better tolerance of treatment toxicity, and lack of competing morbidities (36). Our multivariate analyses of RER also suggest better survival in younger patients even after adjustment for disease stage. This population was the only age group to exhibit no significant excess risk of early mortality 5 years after diagnosis.

One notable finding was a dramatic increase in the 5-year CRS in patients diagnosed in their 70’s. This ‘cross’ phenomenon was also observed in the 3-year disease-free survival of patients aged ≥65 years in a US study (37). This finding might be due to survivor bias: old patients in poor general condition at diagnosis might have forgone or been declined for cytoreductive surgery or chemotherapy. Those patients probably died within a year or two, so those included in the calculation of the 5-year CRS after 3–5 years would be only the patients in better general condition and with early stage disease at diagnosis. In contrast, all patients aged 40–70 might obtain any treatment available, reducing the 5-year CRS 3–5 years after diagnosis.

Differences in CRS between the stage groups became smaller over time, with the largest improvement noted in those diagnosed at the distant stage. However, patients diagnosed at the distant stage still showed a 5-year CRS of only 60% even 5 years after diagnosis, consistent with previous US studies (18, 38). This finding is mainly due to a lowering risk of recurrence after surviving for a certain amount of time. A US study showed that the 3-year conditional risk of recurrence in patients with stage III–IV disease decreased dramatically to around 30% 3 years after achieving remission (37). However, our multivariate analyses showed that stage remained an important prognostic factor even 2 and 5 years after diagnosis (i.e., no significant decrease in RER by time since diagnosis was observed), consistent with previous studies (16, 37). For example, even patients with distant stage disease at diagnosis who survived 5 years, had a 10 times higher risk of death than patients diagnosed with localized disease. This suggests that delayed mortality after a series of anti-cancer treatments (39), risk of late recurrence (40), and toxic late effects from radiation or chemotherapy can cause death in this population even after long-term survival (41). Therefore, clinicians need to pay attention to such risks in this population, in addition to developing effective strategies for early detection.

Patients with serous adenocarcinoma showed poorer 5-year CRS rates at diagnosis but greater improvement over time after diagnosis. That pattern is quite similar to the one found in the US SEER data in 2008 (18, 37), although the survival estimates were slightly higher in our study. The 5-year CRS for serous adenocarcinoma in our study was 57.4% at diagnosis and 74.0% 5 years after diagnosis, whereas the corresponding estimates in the 2008 US study were 39% and 70%, respectively (18). In contrast, the 5-year CRS rates for mucinous, endometrioid, and clear cell cancers were 75–79% at diagnosis and 90–96% 5 years after diagnosis. Those rates are also similar to those in the US, which were 63–66% and 87–94%, respectively. The slightly higher survival rates in our study might be due to improvements in treatment during the time between the two studies and a suggested survival advantage in Asian populations. Although those rates seem to contradict our finding of higher RERs with mucinous, endometrioid, and clear cell histology than serous histology, the latter was mainly due to the different stage and age distributions among the different histologic types. Unlike serous adenocarcinomas, endometrioid, clear cell, and mucinous adenocarcinomas are generally identified at an early stage (42) and in younger women (**Supplementary Table 3** and **Supplementary Figure 1**), and their overall 5-year CRS rates without consideration of stage tend to be better. However, when stage is considered, they have worse survival — for example, advanced stage mucinous or clear cell adenocarcinoma is less sensitive to platinum-based chemotherapy and showed a worse prognosis than serous adenocarcinomas (43, 44). On the other hand, multivariate analyses showed that the prognostic influence of histological types on CS decreased significantly for mucinous and clear cell cancers and even disappeared for the endometrioid type after patients survived for >5 years. This suggests that the disease characteristics collected at diagnosis do not necessarily reflect the prognosis of long-term survivors, and thus their prognostic impact needs to be updated over time.

Patterns of CRS by treatment largely reflected stage information. For example, those who received surgery only would be those with early stage disease (e.g. FIGO stage IA/IB and low grade) which required only surgical treatment. Those who are considered to have received adjuvant chemotherapy (surgery+ chemotherapy group; e.g. FIGO IC-IIIC), had lower survival, and those who received palliative chemotherapy (chemotherapy only group, e.g. FIGO stage IV) showed the lowest survival; however, the 5-year CRS reached 80 and 75% for these group after 5 years of survival, respectively. While this is not the level which is generally considered as a ‘cure’ (5-year CRS>90%) (45), such updated survival estimates need to be communicated to the patients.

The 5-year RS at the time of diagnosis gradually improved from the 1997–2001 period to the 2012–2016 period, consistent with the US SEER data (13). However, the 5-year CRS was higher in patients who were diagnosed in an earlier year. That might reflect natural selection in past patients, with the longer survival of recent patients reflecting improved therapeutic options. For example, advanced ovarian cancer patients diagnosed in past years might have died early on, so as time progresses after diagnosis, a healthier population of patients would remain (17). On the other hand, similar patients with advanced ovarian cancer diagnosed in recent years might survive for several years while on continued cycles of chemotherapy, allowing them to enter into the denominator in calculating CRS rates and decreasing CRS.

Living in a socially deprived area was associated with a lower 5-year RS at diagnosis. While the difference in CRS between areas diminished over time and disappeared 2–3 years after diagnosis, relative excess risk remained significantly elevated even after 5 years, indicating that social deprivation negatively affect survival in the long-term. In Korea, where all Korean people are covered by the national health insurance, surgery and cytotoxic chemotherapy are generally affordable. However, target agents are often paid out-of-pocket, and the poorest people with advanced ovarian cancer might not have been able to afford them, explaining their lower 5-year CRS in the 1–2 years after diagnosis. It is also probable that those living in socially deprived areas receive treatment in low-volume centers, which are associated with higher recurrence and poor survival (46). A similar disparity by socioeconomic status or living area was reported in the US SEER data (38, 47).

Our study strengths include the use of data from a robust national cancer registry with a high level of completeness and a universal health care system. However, this study also has several limitations. First, the KCCR does not contain information on tumor grade, which is important in clinical practice. However, it is considered neither a robust independent prognostic indicator nor a reliable metric for population-based cancer epidemiology research (48). Second, the KCCR does not include information about disease recurrence, so we were unable to analyze conditional disease-free survival, which is also a relevant indicator for survivors and clinicians. Third, KCCR lacks some pathological parameters such as lymph node involvement (49, 50) or BRCA mutation status (51, 52), which may have a significant influence on prognosis. Fourth, KCCR data do not contain information about income, educational status, treatment received, smoking status, menopausal status, or body mass index, which could affect survival probabilities, so we could not examine the effects of those demographic and clinical variables. Fifth, CRS model do not allow for multivariate analyses and cannot account for covariates. We sought to overcome this in part by conducting stratified analyses with various patient characteristics, and by providing RER estimates from multivariate analyses. Fifth, our data might not be generalizable to other healthcare settings because Korea has a universal health insurance system and a free national cervical cancer screening program (53). It is quite common for women to receive an opportunistic ovarian cancer screening *via* transvaginal ultrasound while they are getting the Papanicolaou test for cervical cancer. While ovarian cancer screening has not been proven to be effective, it is possible that increasing early detection through such programs can reduce mortality (54).

## Conclusion

In conclusion, the CRS rates for patients with ovarian cancer improve over time, but they do not reach the level of no excess mortality risk even 5 years after diagnosis. The largest improvements in CRS were observed in patients with poorer initial prognoses, i.e., those who were older, had a higher cancer stage, or serous histology. However, at the same time, these subgroups of patients have 5-year CRS rates <90%, and would require continued surveillance and care. Our study provides useful survival estimates for both patients and healthcare providers based on a patient’s evolving risk profile.

## Data Availability Statement

The original contributions presented in the study are included in the article/**Supplementary Material**. Further inquiries can be directed to the corresponding authors.

## Author Contributions

Conceptualization. K-WJ, DWS, JB. Data curation. K-WJ, JH. Formal analysis: K-WJ, JH. Funding acquisition: K-WJ, Writing. DWS, JB. All authors contributed to the article and approved the submitted version.

## Funding

This work was supported by research grants from the National Cancer Center (1910131). The funding source played no role in the study’s design, data collection and analysis, writing of the report, or decision to submit the report for publication.

## Conflict of Interest

The authors declare that the research was conducted in the absence of any commercial or financial relationships that could be construed as a potential conflict of interest.

## Supplementary Material

Supplementary Figure 1 Histology and stage distribution of ovarian cancer patients **(A)** histology distribution by age group **(B)** stage distribution by histologic type.
