# Supplementary figures and images for: Conditional Relative Survival of Ovarian Cancer: A Korean National Cancer Registry Study
Source: Front Oncol. 2021 Apr 28;11:639839. doi: 10.3389/fonc.2021.639839 (PMC8113866; doi:10.3389/fonc.2021.639839)

Supplementary Figure 1. Distribution of ovarian cancer by age, histology, and stage


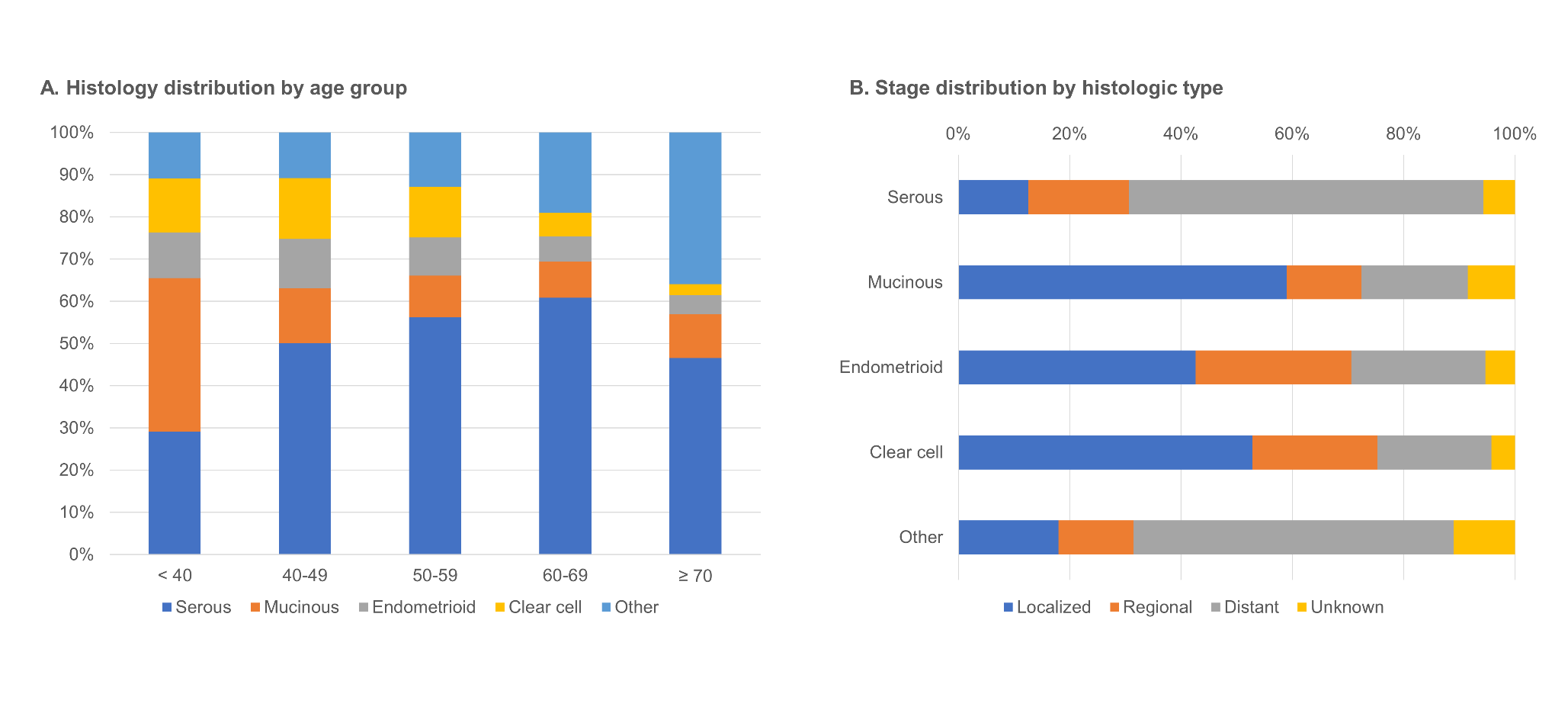

Supplement: Supplementary Figure 1 — Histology and stage distribution of ovarian cancer patients (A) histology distribution by age group (B) stage distribution by histologic type. [file DataSheet_1.docx]
