# Supplementary material for: Conditional Relative Survival of Ovarian Cancer: A Korean National Cancer Registry Study
Source: Front Oncol. 2021 Apr 28;11:639839. doi: 10.3389/fonc.2021.639839 (PMC8113866; doi:10.3389/fonc.2021.639839)
Supplement: Supplementary file 2 [file Table_1.docx]

Supplementary Table 1. Completeness of stage information: collected since 2005.

| Years of diagnosis | Stage |  |  |  |  | Total cases |
| --- | --- | --- | --- | --- | --- | --- |
|  | Localized | Regional | Distant | Unknown | Missing |  |
| 1997 | 0 | 0 | 0 | 0 | 778 | 778 |
| 1998 | 0 | 0 | 0 | 0 | 864 | 864 |
| 1999 | 0 | 0 | 0 | 0 | 925 | 925 |
| 2000 | 0 | 0 | 0 | 0 | 925 | 925 |
| 2001 | 0 | 0 | 0 | 0 | 939 | 939 |
| 2002 | 0 | 0 | 0 | 0 | 990 | 990 |
| 2003 | 0 | 0 | 0 | 0 | 1,046 | 1,046 |
| 2004 | 0 | 0 | 0 | 0 | 1,056 | 1,056 |
| 2005 | 269 | 156 | 463 | 234 | 0 | 1,122 |
| 2006 | 331 | 186 | 505 | 191 | 0 | 1,213 |
| 2007 | 387 | 224 | 645 | 132 | 0 | 1,388 |
| 2008 | 361 | 259 | 630 | 118 | 0 | 1,368 |
| 2009 | 365 | 256 | 659 | 83 | 0 | 1,363 |
| 2010 | 411 | 280 | 780 | 42 | 0 | 1,513 |
| 2011 | 410 | 278 | 746 | 72 | 0 | 1,506 |
| 2012 | 464 | 301 | 815 | 70 | 0 | 1,650 |
| 2013 | 426 | 334 | 830 | 70 | 0 | 1,660 |
| 2014 | 512 | 330 | 885 | 74 | 0 | 1,801 |
| 2015 | 478 | 335 | 893 | 70 | 0 | 1,776 |
| 2016 | 488 | 357 | 1,048 | 83 | 0 | 1,976 |
| 2005-2016 | 4,902 | 3,296 | 8,899 | 1,239 | 0 | 18,336 |
| 1997-2016 | 4,902 | 3,296 | 8,899 | 1,239 | 7,523 | 25,859 |
