# Supplementary material for: Conditional Relative Survival of Ovarian Cancer: A Korean National Cancer Registry Study
Source: Front Oncol. 2021 Apr 28;11:639839. doi: 10.3389/fonc.2021.639839 (PMC8113866; doi:10.3389/fonc.2021.639839)
Supplement: Supplementary file 3 [file Table_2.docx]

**Supplement Table 2. Frequency of histology groups among ovarian cancers by age group and stage, 2005–2016**

|  | **Serous** | | **Mucinous** | | **Endometrioid** | | **Clear cell** | | **Other** | | **Total** | |
| --- | --- | --- | --- | --- | --- | --- | --- | --- | --- | --- | --- | --- |
|  | Cases | % | Cases | % | Cases | % | Cases | % | Cases | % | Cases | % |
| **Age** |  |  |  |  |  |  |  |  |  |  |  |  |
| < 40 | 700 | 7.5 | 876 | 34.1 | 260 | 16.2 | 308 | 16.4 | 262 | 8.7 | 2,406 | 13.1 |
| 40–49 | 2,319 | 25.0 | 603 | 23.5 | 540 | 33.7 | 671 | 35.7 | 500 | 16.6 | 4,633 | 25.3 |
| 50–59 | 3,032 | 32.7 | 537 | 20.9 | 486 | 30.3 | 650 | 34.6 | 695 | 23.1 | 5,400 | 29.5 |
| 60–69 | 2,048 | 22.1 | 289 | 11.2 | 203 | 12.7 | 186 | 9.9 | 641 | 21.3 | 3,367 | 18.4 |
| ≥ 70 | 1,178 | 12.7 | 264 | 10.3 | 114 | 7.1 | 64 | 3.4 | 910 | 30.3 | 2,530 | 13.8 |
| Total | 9,277 | 100.0 | 2,569 | 100.0 | 1,603 | 100.0 | 1,879 | 100.0 | 3,008 | 100.0 | 18,336 | 100.0 |
| **Stage** |  |  |  |  |  |  |  |  |  |  |  |  |
| Localized | 1,170 | 12.6 | 1,516 | 59.0 | 683 | 42.6 | 993 | 52.8 | 540 | 18.0 | 4,902 | 26.7 |
| Regional | 1,675 | 18.1 | 345 | 13.4 | 448 | 27.9 | 422 | 22.5 | 406 | 13.5 | 3,296 | 18.0 |
| Distant | 5,908 | 63.7 | 490 | 19.1 | 387 | 24.1 | 384 | 20.4 | 1,730 | 57.5 | 8,899 | 48.5 |
| Unknown | 524 | 5.6 | 218 | 8.5 | 85 | 5.3 | 80 | 4.3 | 332 | 11.0 | 1,239 | 6.8 |
| Total | 9,277 | 100.0 | 2,569 | 100.0 | 1,603 | 100.0 | 1,879 | 100.0 | 3,008 | 100.0 | 18,336 | 100.0 |
