# Supplementary material for: Conditional Relative Survival of Ovarian Cancer: A Korean National Cancer Registry Study
Source: Front Oncol. 2021 Apr 28;11:639839. doi: 10.3389/fonc.2021.639839 (PMC8113866; doi:10.3389/fonc.2021.639839)
Supplement: Supplementary file 4 [file Table_3.docx]

**Supplementary Table 3. Stage at diagnosis and 5-year relative survival in the US SEER registry (2010-2016) and Korean Central Cancer Registry (2005-2016)**

|  | SEER (2010-2016) | KCCR (2005-2016) |
| --- | --- | --- |
| Stage at diagnosis | % | % |
| Localized | 16 | 26.7 |
| Regional | 21 | 18.0 |
| Distant | 58 | 48.5 |
| Unknown | 9 | 6.8 |
| 5-year relative survival | % | % |
| Localized | 92.6 | 90.7 |
| Regional | 74.8 | 74.7 |
| Distant | 30.2 | 41.7 |
| Unknown | 25.5 | 56.6 |
