# Supplementary material for: Conditional Relative Survival of Ovarian Cancer: A Korean National Cancer Registry Study
Source: Front Oncol. 2021 Apr 28;11:639839. doi: 10.3389/fonc.2021.639839 (PMC8113866; doi:10.3389/fonc.2021.639839)
Supplement: Supplementary file 5 [file Table_4.docx]

**Supplement Table 4. Stage distribution by age groups in Korean ovarian cancer patients, 2005–2016**

| Age at diagnosis, years | < 40 | | 40-49 | | 50-59 | | 60-69 | | ≥ 70 | | Total | |
| --- | --- | --- | --- | --- | --- | --- | --- | --- | --- | --- | --- | --- |
|  | N | % | N | % | N | % | N | % | N | % | N | % |
| Stage |  |  |  |  |  |  |  |  |  |  |  |  |
| Localized | 1,221 | 50.7 | 1,392 | 30.0 | 1,308 | 24.2 | 584 | 17.3 | 397 | 15.7 | 4,902 | 26.7 |
| Regional | 393 | 16.3 | 910 | 19.6 | 1,086 | 20.1 | 568 | 16.9 | 339 | 13.4 | 3,296 | 18.0 |
| Distant | 619 | 25.7 | 2,061 | 44.5 | 2,685 | 49.7 | 1,975 | 58.7 | 1,559 | 61.6 | 8,899 | 48.5 |
| Unknown | 173 | 7.2 | 270 | 5.8 | 321 | 5.9 | 240 | 7.1 | 235 | 9.3 | 1,239 | 6.8 |
| Total | 2,406 | 100.0 | 4,633 | 100.0 | 5,400 | 100.0 | 3,367 | 100.0 | 2,530 | 100.0 | 18,336 | 100.0 |
